# Surface hypothermia predicts murine mortality in the intragastric *Vibrio vulnificus* infection model

**DOI:** 10.1186/s12866-017-1045-z

**Published:** 2017-06-19

**Authors:** Hannah E. Gavin, Karla J. F. Satchell

**Affiliations:** Department of Microbiology-Immunology, Northwestern University Feinberg School of Medicine, 303 E. Chicago Ave, Ward 6-205, Chicago, IL 60611 USA

**Keywords:** *Vibrio vulnificus*, Enteric pathogen, Humane endpoint, Surface temperature, Infrared thermometry

## Abstract

**Background:**

The Gram-negative bacterium *Vibrio vulnificus* can cause severe disease in humans who consume undercooked, contaminated seafood. To study food-borne *V. vulnificus* disease in the laboratory, mouse virulence studies predominantly use death as the primary experimental endpoint because behaviorally based moribund status does not consistently predict lethality. This study assessed ventral surface temperature (VST) and its association with mouse survival during *V. vulnificus* virulence studies as an efficacious, humane alternative.

**Methods:**

VST of mice intragastrically inoculated with *V. vulnificus* was measured every 2-h for 24 h and data for minimal VST analyzed for prediction of lethal outcome.

**Results:**

In contrast to the relatively stable VST of mock-infected control animals, mice infected with *V. vulnificus* exhibited hypothermia with minima occurring 8 to 12 h post-inoculation. The minimum VST of mice that proceeded to death was significantly lower than that of surviving mice. VST ≤ 23.5 °C was predictive of subsequent death with a sensitivity of 68% and specificity of 95%.

**Conclusions:**

Use of VST ≤ 23.5 °C as an experimental endpoint during *V. vulnificus* infection has potential to reduce suffering of nearly 70% of mice for a mean of 10 h per mouse, without compromising experimental efficacy. Temperature cutoff of 23.5 °C exhibited 93% positive and 77% negative predictive value. For future *V. vulnificus* virulence studies requiring only binary comparison (e.g., LD_50_ assays), we find that VST can be applied as a humane endpoint. However, use of VST is not recommended when detailed survival kinetics are desired.

## Background

*Vibrio vulnificus* is a Gram-negative bacterium found in aquatic environments. It has the potential to cause disease in humans who encounter the bacterium [1, 2]. Wound infections occur when open skin lesions are exposed to bacteria, usually during wading or swimming [3]. Gastrointestinal infections arise when humans consume raw or undercooked seafood – most frequently, shellfish – that contains the microbe [4]. In severe situations, these infections progress to necrotizing fasciitis and primary sepsis, respectively [5]. Survival outcomes are particularly poor for gastrointestinal *V. vulnificus* infections with mortality rates exceeding 50% of infected individuals [6]. In light of this infection severity, improved understanding of *V. vulnificus* pathogenesis is increasingly critical.

Controlled human case studies cannot be conducted due to low *V. vulnificus* treatment efficacy, and retrospective clinical analyses lack much of the detailed information necessary to obtain full comprehension of disease course [7–11]. Therefore, to study *V. vulnificus* foodborne infection, a mouse model is routinely employed [12, 13]. Following intragastric inoculation with a bacterial suspension, infected animals are monitored, usually over the course of 48–60 h, by which point they either succumb to or resolve the infection. In lethal cases, bacteria can be isolated from distal organs pre- and post-mortem, indicating this model recapitulates the bacterial dissemination observed in septic human disease [12, 14, 15].

While laboratory mouse experiments provide invaluable data on infectious diseases, animal distress is, in many cases, an inherent component of animal experimentation. For this reason, scientists as well as regulatory bodies have sought methods to limit suffering of murine research subjects [16]. Current data indicate that hypothermia is efficacious for predicting death due to late-stage infectious disease [17–20]. In addition, contact-free, infrared surface thermometers – as an alternative to rectal probe or implanted telemetric thermometers – are ideal for use in studies of acute gastrointestinal pathogenesis. Yet, notably, the specific temperature boundaries that predict infection lethality, and the utility of these boundaries, depend heavily upon the model system in question [17, 21]. Therefore, this study was conducted to test the efficacy of hypothermia as a predictor of *V. vulnificus* mortality after oro-gastric inoculation, and to define the specific temperature parameters for implementation of hypothermia as a humane endpoint in *V. vulnificus* intragastric virulence studies.

## Methods

### Bacterial strain selection

**Fig. 1:**
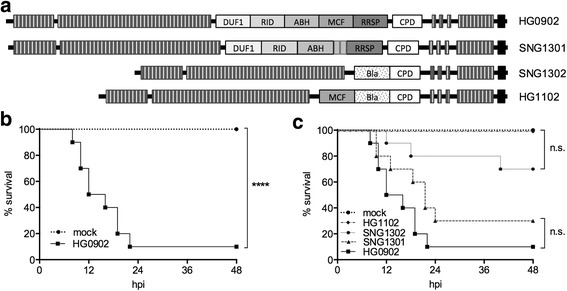
**a** Schematic representation of the MARTX toxin variant produced by the *V. vulnificus* strain name indicated at right. **b** Survival of mice infected with HG0902 compared to mock-infected mice, *n* = 10/group. **c** Survival of mice infected with the indicated strains, *n* = 10/group (note that mock and HG0902 curves are repeated from 1B). Survival curves were compared by log-rank test. ****p ≤ 0.0001

These experiments were conducted using four strains of *V. vulnificus* that differ in the gene *rtxA1*, which encodes the primary virulence toxin, the Multifunctional Autoprocessing Repeats-in-Toxins (MARTX) toxin. All strains are detailed in Table 1 and the toxin variants depicted schematically in Fig. 1a.

**Table 1:** Strains used in this study

	Name	Description	Reference
1	HG0902	CMCP6*rif* Ω*vvhA::bla*	[12]
2	HG1102	HG0902 with *rtxA1::bla*	This study
3	SNG1301	HG9092 with *mcf* deletion in *rtxA1*	This study
4	SNG1302	HG1102 with *mcf* reintroduced	This study

*V. vulnificus* strain HG0902 (CMCP6*rif ΩvvhA::bla*) was generated from the Korean clinical isolate *V. vulnificus* CMCP6*rif*. Although originally described as a genetic deletion of *vvhBA* [12], subsequent gene mapping revealed disruption of only *vvhA* by the stable integration of a portion of ampicillin-resistant plasmid pHGJ4. This strain was then further modified. Plasmids for alteration of *rtxA1* to *rtxA1::bla*, *mcf::bla,* or *Δmcf* have been described previously [15, 22, 23]. Each plasmid was transferred from *Escherichia coli* SM10λ*pir* or S17λ*pir* to *V. vulnificus* HG0902 by conjugation. Selection for double homologous recombination was conducted using sucrose counterselection to isolate recombinants as previously described [24]. Genetic modification was confirmed by polymerase chain reaction (PCR).

### Bacterial growth and preparation

Bacteria were routinely grown in Luria-Burtani (LB) broth (10 g tryptone, 5 g yeast extract, 5 g NaCl) containing 50 μg/mL rifampin as needed. For all experiments, *V. vulnificus* was streaked from frozen glycerol stocks onto LB*rif* plates 2 days prior to experimentation. The following day, single colonies were picked and grown in 2 mL LB broth with selective antibiotic overnight at 30 °C with shaking at 225 rpm. The following morning, strains were subcultured 1:100 into LB without antibiotic and grown to mid-log phase at 30 °C with shaking at 225 rpm. Cultures were pelleted and resuspended in sterile phosphate buffered saline (PBS, 10 mM sodium phosphate, 140 mM NaCl, pH 7.4) to indicated concentrations based on optical density (A_600_). Bacterial inoculum concentration was confirmed by dilution plating.

### Mouse infection

Fifty female C57BL/6 mice were obtained from Jackson Laboratories (Bar Harbor, ME). Animals were housed 5 per cage in wood shavings with disposable paper huts and cotton bedding squares as nesting material. Water and food were provided *ad libitum* *.* Experiments were conducted when mice were 5–6 weeks of age. For each of the two independent experiments, 25 mice were divided into 5 groups of 5 mice each to be either mock-inoculated with PBS, or inoculated with one of the four *V. vulnificus* strains.

Each mouse was transiently anaesthetized using isoflurane and then inoculated intragastrically (i.g.) with 50 μL of PBS or 1 × 10^8^ colony-forming units (CFU) *V. vulnificus* suspended in PBS using a 1-cm animal feeding needle attached to a 1-mL syringe. Mice were then injected intraperitoneally with 100 μL of a cocktail containing 10 μg/ml ketamine and 2 μg/ml xylazine in PBS to facilitate bacterial infection [25].

Mice were monitored hourly for the first 24 h (h) and subsequently every 4–8 h until 48 h post-infection (hpi). Ventral surface temperature (VST) was measured every 2 h during the first 24 h then once at 40–42 h and once at 47–48 h. Temperature was measured using the non-contact infrared TW2 thermometer (ThermoWorks, Alpine, UT) as recommended by the manufacturer. Thermometer emissivity maintained at the manufacturer’s default setting of 0.95. The TW2 thermometer is accurate +/− 1.0 °C when the object of interest is between 15 and 35 °C.

To obtain temperature measurements, mice were restrained by scruffing. The thermometer was held approximately 6 cm from the mouse ventral side and the beam aimed below the base of the sternum, according to procedures that have previously described practices for obtaining consistent temperature readings [26, 27]. Because mouse temperature can change rapidly upon handling, efforts were made to minimize and standardize handling methods across all mice [26, 28, 29]. As such, a temperature stabilization time of 5–10 s was employed prior to temperature recording.

### Statistical analyses

Statistical analyses were performed as indicated in figure legends using GraphPad Prism 6.0 software.

## Results

### *V. vulnificus* strains show distinct survival patterns

For pooled data across both experiments, all ten of the mock-infected animals survived to 48 hpi (Fig. 1b). In contrast, 90% of mice inoculated with the parental *V. vulnificus* strain HG0902 succumbed to infection within 24 hpi (Fig. 1b), an outcome consistent with previous experiments [12, 15]**.** In strain HG1102, the *rtxA1* gene is altered to replace MARTX toxin effector domains with a heterologous beta-lactamase coding region. All mice infected with HG1102 survived to 48 hpi demonstrating significant virulence attenuation (Fig. 1c). These results are consistent with previous data demonstrating that elimination of the MARTX effector domains attenuated virulence equivalent to an *rtxA1*-null mutant [15]. By contrast, deletion of only the *mcf* coding region in SNG1301 did not significantly attenuate virulence compared to HG0902 (Fig. 1c). Similarly, the addition of *mcf* in strain SNG1302 did not significantly enhance virulence of the attenuated strain HG1102 (Fig. 1c). Therefore, the MCF domain alone is neither necessary nor sufficient for MARTX toxin-associated virulence in *V. vulnificus*.

### VST measurement profiles vary dependent upon survival outcome

**Fig. 2:**
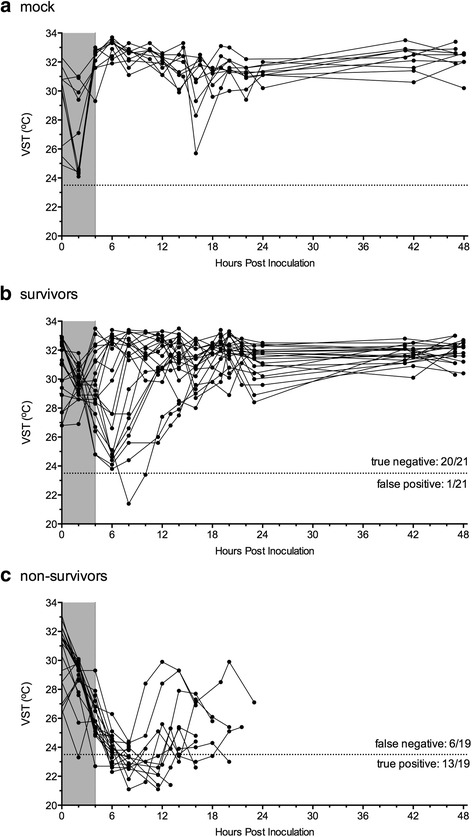
Ventral surface temperature (VST) readings over time for: (**a**) mock infected mice, (**b**) mice that survived infection and (**c**) mice that did not survive infection. Each *dot* represents a single VST reading, and a continuous line connects VST readings from the same mouse. VST measurement ends either at 48 hpi or at time of death

Data were first analyzed pooling results for all *V. vulnificus* groups tested. Of 40 mice inoculated with any strain of *V. vulnificus*, 21 mice survived and 19 mice died. For non-survivors, the mean time-to-death (TTD) was 17 hpi (S.D. ± 8 hpi) with a range of 8 to 40 hpi. In the course of these experiments, the VST was also measured (Fig. 2). Among all mice, the minimum VST (VST_min_) reading during the experiment was 21.1 °C and the maximum VST (VST_max_) was 33.7 °C for a total measurement range of 12.6 °C.

During the first 0 to 4 hpi, the majority (46 of 50) of tested animals exhibited a drop in VST relative to initial (VST_0_). This drop was measured in both infected and mock-treated animals (Fig. 2a-c, grey zones). The temperature decrease at this initial stage is attributed to anesthetic-induced hypothermia [30] as it occurred independent of infection status. For mock-treated animals, VST rebounded to match or exceed VST_0_, usually by 4 hpi and in all cases by 6 hpi (Fig. 2a). VST of mock-infected mice then generally remained stable after recovery from the initial drop (6–48 hpi).

Among *V. vulnificus* inoculated animals, measured VST of mice that survived spanned a wide range of more than 11 °C (Fig. 2b). Survivors most frequently exhibited VST_min_ at 6 or 8 hpi. Subsequent VST readings in survivors were characterized by a period of temperature recovery (8–14 hpi) and finally a period of temperature stability (16–48 hpi).

In contrast, measured VST of mice that succumbed to infection generally did not recover after the anesthetic period of 0–4 hpi (Fig. 2c). Instead, VST of non-survivors continued to decrease, spanned a much smaller range of less than 4 °C, and exhibited VST_min_ readings at 8–12 hpi (Fig. 2c). In many cases, VST of non-survivors increased in the span between 12 and 18 hpi, despite the fact that these animals succumbed to infection by 24 hpi. This pattern is distinct both from the mock-treated group and infected animals that survive inoculation. Thus, VST measurement profiles vary depending upon both *V. vulnificus* infection status, and infection survival outcomes.

### VST of 23.5 °C is associated with lethality during *V. vulnificus* infection

**Fig. 3:**
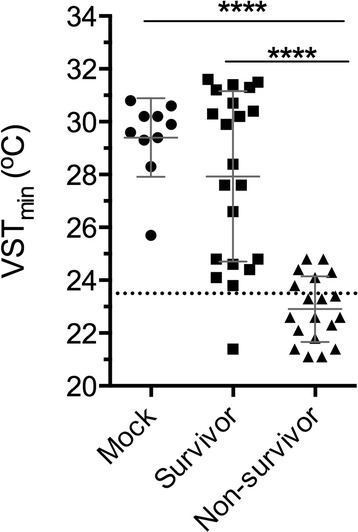
Scatter plot of VST_min_ measured between 6 and 48 hpi. *Lines* indicate mean with error bars for standard deviation and temperature cutoff of 23.5 °C is indicated with a dashed line. Results analyzed by one-way ANOVA with multiple comparisons. ****p ≤ 0.0001

Comparing VST between 6 and 48 hpi across groups, mean VST_min_ of surviving mice (27.9 ± 3.2 °C, Fig. 3) was not significantly different from mean VST_min_ of mock-infected mice (29.4 ± 1.5 °C, Fig. 3). However, mice that later succumbed to infection exhibited significantly reduced VST_min_ compared to either survivors or mock-treated animals (22.9 ± 1.2 °C Fig. 3). Detailed analysis suggests that 23.5 °C delineates between lethally and non-lethally infected mice.

Only 1 of 21 survivors exhibited VST ≤ 23.5 °C (false positive, Fig. 2b). In diagnostic terms, a test’s specificity is its ability to correctly identify individuals that are not affected by a condition. In other words, specificity measures the test’s true negative rate as a ratio of true negatives calls to total negative outcomes. In this case, where survival is the experiment negative outcome, 21 mice survived infection and 20 of those mice had VST_min_ > 23.5. Thus, the endpoint of VST ≤ 23.5 °C exhibits specificity of 95%. This high specificity result is important because it indicates only 5% of VST-indicated euthanizations will result in a mouse being counted as a non-survivor that otherwise would have survived infection.

Conversely, 13 of 19 non-survivors reached VST ≤ 23.5 °C (true positive, Fig. 2c). The sensitivity of a diagnostic test indicates its ability to correctly identify those individuals affected by a condition in question. Sensitivity measures true positive rate by taking a ratio of true positive calls to total positive outcomes. In the case of VST endpoints applied to *V. vulnificus* infections, where death is the test’s positive outcome, 13 non-survivor mice were predicted by their VST_min_ to have lethal infection, while a total of 19 mice were ultimately non-survivors. Therefore, VST ≤ 23.5 °C has a sensitivity of 68% in predicting *V. vulnificus* infection outcome. This result means that 68% of mice that spontaneously died were indicated by VST ≤ 23.5 °C. Moreover, because the remaining mice with lethal infections still proceed to death, the sensitivity of the overall experiment is actually unchanged; eventually all non-survivor outcomes are captured within the experimental context. Thus, with essentially no loss of experimental efficacy, nearly 70% of mice could have been humanely euthanized for a reduction of suffering prior to the onset of death.

Two additional measures are used to assess the diagnostic efficacy of VST ≤ 23.5 °C. Positive predictive value (PPV) is the proportion of positive diagnostic calls that are, indeed, true positives. In this case, 14 mice reached VST ≤ 23.5 °C. 13 of these 14 mice were non-survivors. Therefore, the hypothermia endpoint has a PPV of 93%. Conversely, the negative predictive value (NPV) is the proportion of negative test calls that are true negatives outcomes. Twenty of the 26 mice that had temperatures above the cutoff were survivors, while 6 were non-survivors. Therefore, the hypothermia endpoint VST ≤ 23.5 °C has a NPV of 77%. This analysis supports the conclusion that reduction in VST is predictive of subsequent death.

### VST ≤ 23.5 °C predicts live/dead survival outcomes and reduces infection hours

**Fig. 4:**
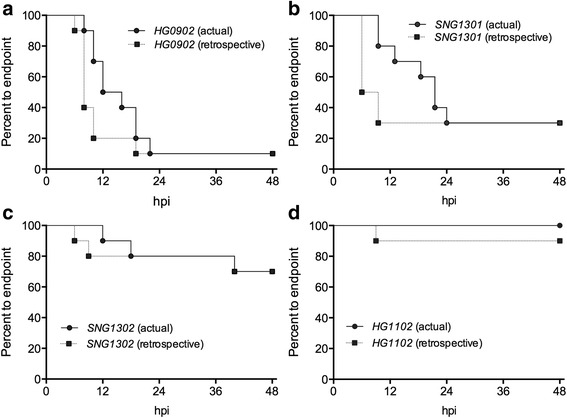
Comparison of survival time for each of the *V. vulnificus* strains: (**a**) HG0902; (**b**) SNG1301; (**c**) SNG1302; (**d**) HG1102. Actual curves (*circles connected by solid line*) use death as endpoint; retrospective curves (*squares connected by dotted line*) use VST ≤ 23.5 °C, or death if an animal died without reaching VST ≤ 23.5 °C. Statistical analyses are summarized in Table 2

The survival data were next analyzed comparing temperature-predicted outcomes to actual outcomes. To assess the efficacy of hypothermia as an experimental endpoint when comparing different infecting bacterial strains, time-to-death and time-to-VST ≤ 23.5 °C were compared for each infecting strain in this study (Fig. 4). In the retrospective analysis (dotted lines), animals were plotted as non-survivors if either (1) the mouse exhibited VST ≤ 23.5 °C or (2) if the mouse died even if it never reached ≤23.5 °C.

Statistical variation of binary outcomes (survival vs. death) was analyzed by chi-square test (χ^2^, Table 2). The χ^2^ analysis revealed no significant differences between binary survival outcomes when the hypothermia plus actual death endpoint was applied (Fig. 4, Table 2). In fact, in most cases the survival outcomes were identical (Fig. 4a-c), but even in the case of a numerically different outcome the results were not significantly different (Fig. 4, Table 2).

**Table 2:** Statistical analyses of survival outcomes and curves

Strain	Binary Χ^2^ Analysis (actual vs. retro)		Log-rank curve comparison (actual vs. retro)	
	χ^2^	*p*	χ^2^	*p*
HG0902	0.0	1.0	1.8	0.18
SNG1301	0.0	1.0	0.82	0.36
SNG1302	0.0	1.0	0.0085	0.92
HG1102	1.05	0.30	1.0	0.32

VST ≤ 23.5 °C in some cases correlated with imminent death, although many animals lived for hours prior to succumbing to infection (Fig. 2c). In non-survivors, TTD after VST ≤ 23.5 °C had a range of 15 h, mean of 10 h, and S.D. of 5 h. Therefore, while this endpoint is capable of predicting a lethal outcome, there is notable variability in the TTD. To illustrate this point, the endpoint curves (Fig. 4) were compared using the log-rank test with 95% confidence, *p* < 0.05. Interestingly, there were no statistical differences between endpoint curves when analyzed by log-rank test (Table 2). Nonetheless, detailed information on the kinetics of death were lost in the hypothermia-derived curves due to time discrepancies between VST ≤ 23.5 °C and actual death (Fig. 4). While this renders the VST endpoint unsuitable for survival curve comparison, the differences in area under the survival curves (Fig. 4) illustrates the power of the alternative VST endpoint to perform its intended function: predict non-survivor outcomes, such that animals involved in the experiments can be euthanized prior to the onset of death. In the experiments illustrated in Fig. 4, the hypothermia endpoint VST ≤ 23.5 °C would have eliminated 135 unnecessary infection hours for mice that would eventually succumb to infection (dotted vs solid lines).

## Discussion

Measurement of virulence in animal models is central to studies of bacterial pathogenesis. In lieu of death, the ‘moribund condition’ – generally characterized by markers such as piloerection, hunching, and respiratory irregularity – is often utilized in animal protocols as an experimental endpoint, considered more humane than death. However, moribund status can be biased due to subjectivity and inconsistency of observers, and inaccurate if there is disconnect between observed characteristics and imminent death [21, 31]. In our research group, we have noted anecdotally that there is a lack of correlation between moribund status and imminent death during *V. vulnificus* experiments. In the past 25 years, temperature monitoring has been successfully utilized to develop more refined endpoints for fungal, bacterial, and viral infection studies [19, 27, 32–34]. Retrospective analysis of additional infectious disease experiments found hypothermia to be “the most valuable characteristic for distinguishing mice that survive or succumb to infection” [17].

Core body temperature can be measured by rectal thermometry [19, 20], however, this method is time consuming for research staff and is thought to increase animal distress due to extensive handling [21, 26]. Moreover, intestinal probing has the potential to adversely affect experimental outcomes of a gastrointestinal pathogen due to the risk of lesion introduction [35]. The alternative method of microchip implants for thermal telemetry facilitates rapid, contact-free measurement by researchers, but requires front-end time and monetary output that is not justifiable for the acute time scale of *V. vulnificus* infections [18, 21, 36]. In addition, lesions generated at the microchip injection site are susceptible to unintentional infection [26]. Since *V. vulnificus* infects open wounds [3], the risk of subcutaneous infection is a confounding factor that renders microchip thermometry untenable for *V. vulnificus* studies.

With the advent of new technology, body surface temperature can now be measured using an external infrared thermometer [26, 27, 34]. Compared to other methods, use of a surface probe reduces animal stress, experimental complication, and initial cost [37]. Importantly, measured core and surface temperatures are correlated [37]. Correlations are particularly strong when core body temperatures are below normal, as is the case during infection-induced hypothermia [17, 37]. In the current study, infrared surface thermometry demonstrated utility in predicting disease severity during *V. vulnificus* infection and was utilized to identify a hypothermic temperature endpoint for these studies.

This study was conducted using four strains of *V. vulnificus* that differ in their known or predicted virulence. Predicted or realized differences in virulence were derived from modification of the virulence associated gene *rtxA1.* Indeed, when the central portion of the toxin – collectively termed the effector domain region – was deleted and replaced by a heterologous beta-lactamase coding sequence, *V. vulnificus* was significantly less virulent (Fig. 1c, HG0902 vs HG1102, [15]). To examine the necessity of a single effector domain in virulence we tested a strain SNG1301 that harbors an in-frame deletion of the *mcf* effector domain (Fig. 1a) [23] and another that carries only the *mcf* effector domain region. These experiments support the conclusion that the *mcf* coding region is neither necessary nor sufficient to change the virulence potential of *V. vulnificus*. These data provided a virulence data set as a baseline for assessing the use of VST as an endpoint for *V. vulnificus* intestinal infections.

In many cases, differences in pathogen virulence are best defined by median lethal dose (LD_50_) determination. These studies need not take experimental kinetics into account, because LD_50_ is determined by the number of survivors and non-survivors at a given pathogen dose. In the current study, 68% of mice that spontaneously died could have been euthanized on average 10 h prior to death using the defined VST endpoint. At 95% sensitivity, application of this endpoint did not compromise experimental efficacy. Thus, applying an endpoint of VST ≤ 23.5 °C during *V. vulnificus* infection has potential to considerably reduce animal suffering in studies where binary data is desired. Our finding that binary live vs. dead outcomes are predictable by hypothermia suggests that the VST alternative endpoint is appropriate for LD_50_ determination and should be applied to LD_50_ studies to reduce suffering of animals in these experiments.

LD_50_ measurements require large numbers of mice to test an appropriate range of doses at a level that allows for statistical power. As such, many research groups use an alternative method for determining relative virulence: infecting different groups of mice with different strains at a set dose (as in Fig. 1c) and reporting differences in the survival curves. Unlike binary LD_50_ studies, these curves account for time-to-death, which allows for greater statistical power with lower numbers of mice. Yet, survival curves are also considered less sensitive in their ability to detect virulence differences among strains. This study reveals that the observed lag between the VST endpoint and death renders application of a VST endpoint a poor choice for these types of studies (Fig. 4, actual vs. retrospective) due to loss of death kinetics. The curve shift actually emphasizes the utility of VST ≤ 23.5 °C in predicting lethal outcomes well before the onset of death to the experimental animals. Yet, this result likewise indicates that a VST-based endpoint does not have the same cost-free benefit in survival curve experiments that it offers to LD_50_.

Interestingly, these results leave researchers to determine whether it is more ethical to: (A) use larger numbers of mice and perform LD_50_ studies, with the ability to apply a humane experimental endpoint; or (B) use smaller numbers of mice in a survival curve analysis without the option of a humane endpoint, a decision that will depend largely on the specific experimental goals of the research group in question.

## Conclusions

While hypothermia has been demonstrated as a key marker of death due to infectious disease, its relationship to survival of *V. vulnificus* infections had not previously been examined. It was here determined that surviving and non-surviving *V. vulnificus* infected mice demonstrate distinct temperature responses. Non-surviving infected mice have significantly lower VST_min_ as compared to surviving mice. From these data, a temperature endpoint of 23.5 °C was empirically determined and retrospectively applied to the survival results. When used as an experimental endpoint, VST ≤ 23.5 °C exhibited sensitivity of 68% and specificity of 95%. The temperature cutoff of 23.5 °C demonstrates 93% PPV and 77% NPV. Given the outcomes of this study, a hypothermia-based humane endpoint can be applied to LD_50_ studies without concern of reducing the efficacy of the experiment; binary (live vs. dead) survival outcomes are almost always numerically identical and in all cases statistically indistinguishable from survival data using death as an endpoint (Fig. 4, Table 2). As such, many hours of undue suffering would be eliminated from infection experiments.

By its very nature, an alternative endpoint aims to predict death early such that animal research subjects can be euthanized prior to death. VST ≤ 23.5 °C predicts non-survival outcomes an average of 10 h in advance of the event. This allows for a considerable reduction in unnecessary mouse infection hours. Nonetheless, given that a hypothermia endpoint alters experimental kinetics, these kinetic changes suggest that VST endpoint should not be applied to survival curve experiments.

## Abbreviations

h: Hour(s)
hpi: Hours post infection
LD_50_: Median lethal dose
n.s.: Not significantly different
NPV: Negative predictive value
PPV: Positive predictive value
S.D.: Standard deviation
TTD: Time to death
VST: Ventral surface temperature
